# Adverse childhood experiences and adolescent externalizing and internalizing problems in the ELSPAC-CZ cohort

**DOI:** 10.1186/s13034-025-01004-1

**Published:** 2025-12-24

**Authors:** Gabriela Ksinan Jiskrova, Albert J. Ksinan, Hynek Pikhart, Martin Bobák, Jana Klanova, Rebecca E. Lacey

**Affiliations:** 1 RECETOX, Faculty of Science, Masaryk University, Kamenice 5, 625 00 Brno, Czech Republic; 2 Department of Epidemiology & Public Health, University College London, London, UK; 3 School of Health & Medical Sciences, City St George’s, University of London, London, UK

**Keywords:** ACE score, Adverse childhood experiences, European longitudinal study of pregnancy and childhood, Externalizing, Internalizing

## Abstract

**Background:**

Exposure to adverse childhood experiences (ACEs) has been linked to mental health difficulties later in life. However, much of the existing research relies on cross-sectional designs and retrospectively reported ACEs, which are susceptible to recall bias and confounding by early life factors, such as family socioeconomic status or childhood temperament. Moreover, the majority of these studies have been conducted in the United States and the United Kingdom, limiting the generalizability of their findings. To address these limitations, we examined the association between prospectively measured ACEs and adolescent adjustment using data from a longitudinal, population-based birth cohort in Central Europe.

**Methods:**

Data were obtained from the Czech part of the European Longitudinal Cohort Study of Pregnancy and Childhood (ELSPAC-CZ; *N* = 2,741). ACE score was calculated as a sum of eight intra-familial adversities assessed prospectively between 6 months and 11 years postpartum. Adolescent internalizing and externalizing problems were measured via Strength and Difficulties Questionnaire (SDQ) at 11 years and were reported by adolescents and their mothers.

**Results:**

Linear regression models showed that ACE score was associated with internalizing problems reported by adolescent (β = 0.063, 95% CI [0.019, 0.107]) and mother (β = 0.120, 95% CI [0.077, 0.163]), and externalizing problems reported by adolescent (β = 0.088, 95% CI [0.045, 0.132]) and mother (β = 0.114, 95% CI [0.072, 0.157]). The association was driven particularly by physical and emotional abuse.

**Conclusions:**

ACE were common in ELSPAC-CZ sample (69% of children experienced at least one ACE) and were prospectively associated with adjustment in adolescents, independently from family socioeconomic status, prenatal and birth characteristics, and early childhood temperament, suggesting a robust link between ACE and adolescent adjustment.

## Introduction

Adverse childhood experiences (ACE) are a well-established risk factor for poor physical and mental health later in life [1, 2]. ACE are defined as childhood experiences (before age 18) that exceed developmentally appropriate levels of stress [3, 4]. These experiences are typically conceptualized as child maltreatment and serious forms of household dysfunction [5]. ACE are common; existing studies report that between 47 and 67% of individuals experienced one ACE and between 9 and 16% four or more ACE [6–8]. ACE are costly for society – the annual costs attributable to ACE were estimated to be $581 billion in Europe and $748 billion in north America [9].

ACE were linked to a number of health risk behaviors, and chronic non-communicable diseases [10]. In their systematic review and meta-analysis, Hughes and colleagues [2] showed that compared to individuals who were not exposed to ACE, those who experienced at least four ACE had an increased odds of obesity, diabetes, substance use, heart and respiratory disease, cancer, and mental ill health. The link between ACE and indicators of mental health is particularly salient, with many studies suggesting a graded dose-response relationship [11]. ACE were linked to depression symptoms [12–14], depression recurrence, and lower response to the treatment [15]. Although the link between ACE and mental ill health has been established mostly in adult samples, there is an evidence of similar associations in adolescents and young adults [16–18].

Although the association between ACE and health problems appears well-established, some researchers raised concerns about the robustness of the existing evidence [19–22]. First, most of the existing ACE studies relied on cross-sectional samples with retrospective measures of childhood adversity collected during adulthood. Although important, particularly when done with nationally representative samples, studies utilizing retrospective measures may suffer from recall bias and may produce inflated estimates of the association between ACE and the outcome if reported by the same informant. Indeed, previous studies showed that retrospectively reported childhood adversity is more strongly associated with later psychopathology than prospective measures [23, 24].

Second, in the dominant explanatory model, the health consequences are interpreted as causal outcomes of childhood adversity without considering any preexisting differences between children exposed and not exposed to ACE [21]. To illustrate this point, Danese and colleagues [22] tested the association between ACE and later cognitive functioning using data from two large population-based prospective cohorts with repeated measures of cognitive functioning. The results showed that worse cognitive functioning was largely explained by preexisting cognitive vulnerability and a socioeconomic disadvantage that predated ACE. Although research in this area remains limited, several studies [25–27] have examined the relationship between childhood adversity and later emotional and behavioral functioning while accounting for early emotional vulnerability, often operationalized as difficult temperament—a well-established early predictor of later adjustment problems [28–30]. These studies found that the association between childhood adversity and later outcomes persisted even after controlling for early temperament. Importantly, temperament also independently contributed to the development of emotional and behavioral difficulties.

Third, most of the ACE studies were carried out in the United States and the United Kingdom [2]. While the evidence from other countries is gradually emerging, there is a lack of research on ACE from Central and Eastern Europe. This region has experienced distinct societal transitions and unique socioeconomic challenges, which may shape family characteristics and developmental outcomes. The available studies from the region has focused on validation of measurement tools [31], investigating ACE prevalence [32] or cross-sectional associations between ACE and the outcomes [33, 34]. To the best of our knowledge, no study so far has investigated the effects of ACE in prospective, population-based cohort samples.

The aim of the current study was to investigate the association between ACE and adolescent adjustment (internalizing and externalizing problems) while strengthening the causal inference and addressing concerns raised in the literature about methodological limitations of earlier work. First, we utilized data from a population-based longitudinal birth cohort with prospectively collected ACE to overcome the limitations of previous research based on retrospective and cross-sectional data. Information on adolescent outcomes was reported both by mothers and adolescents to minimize the same-reporter bias. Second, we controlled for a number of socioeconomic and birth characteristics, and early childhood temperament that predated ACE victimization in all analyses. Lastly, this study brings evidence on ACE from Central Europe, an under-represented region in the ACE research.

## Methods

### Sample

Data were obtained from the Czech arm of the European Longitudinal Study of Pregnancy and Childhood (ELSPAC-CZ), a multi-site population-based birth cohort. The study was initiated by the World Health Organization Regional Office for Europe in 1985 and coordinated by Bristol University (ALSPAC) with the aim to collect data across Europe on biological, psychosocial, economic, and environmental predictors of maternal and child health [35, 36]. In ELSPAC-CZ, pregnant women from two metropolitan areas were enrolled in the study between March 1, 1991 and June 30, 1992. Health records about pregnancy and delivery, and questionnaire data were collected at the baseline from *N* = 5,151 mothers/primary caregivers and *N* = 4,653 fathers/partners. Follow-up assessments included medical examinations at 13 timepoints between prenatal period and 19 years of age, and self-reported questionnaires from mothers, their partners, teachers, and children themselves [36]. Informed consent was obtained from all participants. The study was approved by the ELSPAC Law and Ethics Committee and local research ethics committees. The secondary use of all ELSPAC study data was approved by the (C)ELSPAC Ethics Committee (Ref. No. ELSPAC/EK/1/2014, date 09/17/2014).

### Measures

*ACE* Information on ACE was collected prospectively from primary caregivers/mothers, partners/fathers, children themselves, and pediatricians from birth until age 19. As the ELSPAC questionnaires were created by translating the original ALSPAC questionnaires from English to Czech language, we derived the ACE types and ACE score according to the guidelines developed for ALSPAC [37] and consistently with previous studies assessing ACE in ALSPAC cohort [38, 39]. For the purpose of the current study, we defined ACE as eight intra-familial adversities [3] that the child has been exposed to between birth and 11 years, using mother and father-reported data collected at six time points: 6 months, 18 months, 3, 5, 7, and 11 years. The eight ACE types were measured in the following way at each respective time point assessed since the last survey:

*Sexual abuse* was coded as having occurred if the mother responded affirmatively to the item “Your child was sexually abused”. The item was not available at the first assessment point (age 6 months).

*Physical abuse* Children were considered as having been exposed to physical abuse if the mother, father, or both responded affirmatively to any of the following items: “You physically hurt your children”, and “Your partner physically hurt your children”.

*Emotional abuse* Children were considered as having been exposed to emotional abuse if the mother, father, or both responded affirmatively to any of the following items: “You were emotionally cruel to your children”, and “Your partner was emotionally cruel to your children”.

*Parental mental illness* Children were coded as being exposed to parental mental illness if the mother, father, or both: (a) scored 13 or more on the Edinburgh Postnatal Depression Scale, indicating an increased risk of depression disorder [40]; (b) reported a suicide attempt, or (c) reported they consulted a doctor for depression or anxiety.

*Parental offending* was indicated by an affirmative answer by mother, father, or both to any of the following items: a) “You were in trouble with the law”; “Your partner was in trouble with the law”; and c) “You were convicted of an offence”.

*Parental divorce or separation* Children were coded as being exposed to parental divorce or separation if the mother, father, or both: (a) reported a change in the marital status from *married* at earlier time point to *divorced/separated* at later time point or answered affirmatively any of the following questions: (b) “You got divorced”; (c) “Your partner left you”; and (d) “You broke up with your partner”.

*Parental conflict and violence* were indicated by an affirmative answer by mother, father, or both to any of the two following items: (a) “Your partner physically hurt you”, or (b) “Your partner was emotionally cruel to you”.

*Parental substance use* Children were considered as having been exposed to parental substance use if the mother, father, or both reported: (a) own or partner’s daily alcohol consumption of 3 or more drinks; (b) own or partner’s doctor consultations for problems with alcohol; (c) daily marijuana use, or (d) any use of heroin, cocaine, or crack.

*ACE score* was calculated as a sum of the eight different types of ACE reported between ages 3 and 11. The score ranging between 0 and 8, depending on the number of ACE types reported, was then recoded into the following categories: 0, 1, 2, 3, and ≥ 4 ACE. The age range was selected so that ACE victimization temporally follows the measurement of childhood temperament and precedes the assessment of the adolescent adjustment.

*Internalizing and externalizing problems* were assessed by the Czech version of the Strength and Difficulties Questionnaire (SDQ; 42), a measure with established psychometric properties and previously used in the ELSPAC-CZ cohort [42]. Children and their mothers answered 10 items assessing internalizing (e.g., “In the last 6 months, your child was/you were sad or depressed.”) and externalizing (e.g., “In the last 6 months, your child was/you were angry or irritable.”) problems during the past 6 months at age 11 years. The items were answered on a four-point Likert-type scale ranging from *never true* (0) to *always true* [3]. The internalizing and externalizing problems score for both reporters was calculated by summing the respective 10 items part of each subscale (Cronbach’s ɑ range = 0.66 to 0.81).

*Temperament* was measured by the EAS Temperament Survey for Children: Parental Ratings (Buss & Plomin, 1984) at 3 years. Mothers answered twenty items designed to assess four dimensions of children’s temperament: emotionality (e.g., “Your child reacts intensely when upset.”), activity (e.g., “Your child is always on the go.”), sociability (e.g., “Your child likes to be with people.”), and shyness (e.g., “Your child takes a long time to warm up to strangers.”). The dimension sociability was strongly correlated with shyness (*r* = -.54) and activity (*r* = -.44) as reported also by Boer and Westenberg [43]. Thus, we decided to exclude this dimension from the analysis. The items were answered on a five-point Likert-type scale ranging from *not characteristic or typical of your child* (0) to *very characteristic or typical of your child* [4]; (ɑ range = 0.68 to 0.82).

*Maternal prenatal depressive symptoms* were assessed by the Edinburgh Postnatal Depression Scale, an instrument validated for pregnancy and postpartum (EPDS; [41]) by mother-reported questionnaire administered in mid-pregnancy. Mothers answered 10 self-reported items on a four-point Likert-type scale ranging from *never* (0) to *most of the time* [3]. The depressive symptoms score was calculated by summing the items part of the measure (ɑ = 0.82).

*Alcohol use in early to mid-pregnancy* was assessed by two items asking about frequency of drinking in the first trimester and around the time of baby’s first movements. The frequency was reported on a four-point scale ranging from *never* = 0 to *more than 1–2 servings per day* = 3 at mid-pregnancy. Mothers were coded as having consumed alcohol if they reported *ever* [1] versus *never* (0) consuming alcohol.

*Smoking in pregnancy* was assessed by two mother-reported items asking whether the mother smoked and if so whether she stopped during the pregnancy asked at mid-pregnancy. The mothers were coded as *non-smokers* (0) if they never smoked, *ex-smokers* [1] if they stopped during the pregnancy, and *smokers* [2] if they did not stop during pregnancy.

*ACE score by 3 years* was calculated as a sum of eight different types of ACE reported between birth and 3 years. The score ranging between 0 and 8 was recoded into the following categories: 0, 1, 2, 3, and ≥ 4 ACE. The score was used as a covariate to control for possible effects of ACE victimization that occurred before age 3.

*Demographic variables* included maternal age at delivery (years), family structure (*two biological parents* = 1; *other* = 0) assessed at 6 months after birth, maternal education level prior to birth (ranging from *elementary school* = 1 to *college degree* = 5) and a mother-reported financial hardship at 6 months assessing how difficult it was to provide items for the family including food, clothing, heating, rent/mortgage payments, and things for the child. Each item was rated on a four-point scale (*not difficult* = 0; *very difficult* = 3); the items were averaged to calculate the total scale score (ɑ = 0.84).

*Delivery characteristics* included child sex (*male* = 1; *female* = 0) and birth weight coded as *low* (< 2,500 g = 1) and *normal* (≥ 2,500 g = 0) obtained from medical records. Parity was coded based on mother’s reports collected in mid-pregnancy on whether they already had children (*nulliparous* = 1; *multiparous* = 0).

### Plan of analysis

For the purpose of the current study, we limited the initial sample (*N* = 5,151) to participants who had data available for at least one of the four outcomes assessed at age 11 (i.e., adolescent and mother-reported internalizing and externalizing problems), resulting in an analytical sample of *N* = 2,741. Missing data were handled using multiple imputation with the R package *mice* [44]. All predictor and outcome variables were included in the imputation model. A total of 50 imputed datasets were created using predictive mean matching (PMM) for continuous variables and logistic or polytomous regression for categorical variables. Each dataset was imputed for 50 iterations to ensure convergence. All analyses were performed on each imputed dataset separately, and results were combined using Rubin’s rules to obtain pooled estimates and standard errors.

Attrition analysis comparing the analytic sample to the full sample on baseline variables showed that participants not included in the analytic sample had lower levels of maternal education (e.g. 10.1% elementary school among excluded vs. 5.2% among included, *p* < .001), higher proportion of smoking during pregnancy (10.1% excluded vs. 6.6% included, *p* < .001), higher proportion of ex-smokers (36% excluded vs. 31.5% included, *p* < .001), lower proportion of two-parent family structure at 6 months (88.3% excluded vs. 91.8% included, *p* < .001), lower birth weight (5.9% excluded vs. 4.3% included, *p* = .002), and higher proportion of males among children (52.6% excluded vs. 50% included, *p* = .030). Other baseline variables, such as parity, prenatal depression, alcohol use in pregnancy, or prenatal depression were not significantly different between groups.

Next, a series of hierarchical linear regression models was computed to examine the associations between ACE and the four outcomes, i.e. adolescent-reported internalizing and externalizing problems and mother-reported internalizing and externalizing problems. First, we tested whether ACE score was related to each of the four outcomes (Model 1); second, we adjusted the model for socioeconomic and birth characteristics (Model 2), and third, we included the temperament dimensions into the model (Model 3). The models were run separately for each of the four outcomes. Lastly, we tested the associations between the eight ACE types and the outcomes separately, adjusting for all covariates. Benjamini-Hochberg false discovery rate (FDR) correction was applied for this analysis.

Follow-up mediation and moderation analyses were performed to further disentangle the associations between ACE, early childhood emotionality, and adjustment. First, we specified four moderation models that included an interaction term between difficult temperament and ACE, along with their main effects and covariates. We focused on the *emotionality* dimension of temperament as it best captures the core of difficult temperament—namely, the tendency to react with negative emotions to various stimuli [52]. Each outcome was tested in a separate model, resulting in four moderation analyses. Second, we specified four mediation models to test whether ACE scores between ages 3 and 11 mediated the association between emotionality at age 3 and the adjustment outcomes. Again, one model was tested for each outcome.

Multicollinearity tests did not provide evidence of any problems (all VIF estimates < 2.00). The regression models were estimated using imputed data and the reported results are pooled estimates. Statistical analyses were conducted using M*plus* 8 software [45] using maximum likelihood with robust standard errors (MLR) estimator to account for the deviations from normality in variable distributions. A complete case analysis was conducted for all the models to compare the results based on the analytical and imputed dataset. All estimates were similar in magnitude; the results of the complete case analysis are available upon request.

## Results

Descriptive statistics of the study variables for the analytical and imputed sample are summarized in Table 1.

**Table 1:** Descriptive statistics of study sample

	Analytical		Imputed *N* = 2,741
	*N*	Mean/%	Mean/%
ACE type			
Sexual abuse	2,738		
Yes	79	2.9	5.0
No	2,659	97.1	95.0
Physical abuse	2,737		
Yes	418	15.3	17.2
No	2,319	84.7	82.8
Emotional abuse	2,737		
Yes	483	17.6	19.9
No	2,254	82.4	80.1
Parental mental illness	2,737		
Yes	1,034	37.8	39.7
No	1,703	62.2	60.3
Parental offending	2,737		
Yes	261	9.5	11.6
No	2,476	90.5	88.4
Parental divorce or separation	2,737		
Yes	570	20.8	22.6
No	2,167	79.2	77.4
Parental conflict and violence	2,737		
Yes	1,709	37.6	39.6
No	1,028	62.4	60.4
Parental substance use	2,737		
Yes	336	12.3	13.9
No	2,401	87.7	86.1
ACE score	2,741		
0	914	33.3	30.7
1	696	25.4	25.0
2	459	16.7	16.5
3	305	11.1	12.0
4+	367	13.4	15.9
SDQ			
Internalizing problems – adolescent	2,530	7.4	7.4
Internalizing problems – mother/caregiver	2,544	5.7	5.7
Externalizing problems – adolescent	2,532	8.9	8.9
Externalizing problems – mother/caregiver	2,543	7.5	7.5
Covariates			
Child sex	2,741		
Male	1,371	50.0	50.0
Female	1,370	50.0	50.0
Birth weight	2,678		
< 2,500 g	114	4.3	4.3
≥ 2,500 g	2,568	95.7	95.7
Maternal age (years)	2,730	25.4	25.4
Parity	2,183		
Nulliparous	1,093	50.1	50.3
Multiparous	1,090	49.9	49.7
Maternal education	2,198		
Elementary school	114	5.2	5.6
Trade school	576	26.2	26.4
High school diploma	909	41.4	40.6
Some college	123	5.6	5.5
College degree	476	21.7	21.9
Maternal prenatal depressive symptoms	2,206	6.4	6.9
Alcohol use in early to mid-pregnancy	2,114		
Yes	731	34.6	37.4
Never	1,383	65.4	62.6
Smoking in pregnancy	2,192		
Non-smokers	1,357	61.9	60.2
Ex-smokers	690	31.5	31.9
Smokers	145	6.6	7.9
Financial hardship	2,608	0.6	0.6
Family structure	2,199		
Two biological parents	2,018	91.8	90.5
Other	181	8.2	9.5
ACE score by 3 years	2,732		
0	1,142	41.8	36.5
1	746	27.3	26.1
2	414	15.2	15.0
3	212	7.8	8.1
4+	218	8.0	14.3
Activity	2,407	21.1	20.7
Emotionality	2,398	14.8	14.9
Shyness	2,427	11.6	12.0

All percentages are valid percent

Regarding the outcome, adolescents themselves tended to report higher levels of both internalizing (*M* = 7.4 vs. *M* = 5.7, *p* < .001) and externalizing problems (*M* = 8.9 vs. *M* = 7.5, *p* < .001) than what their mothers reported about them. The correlation coefficients between adolescent and maternal reports were 0.41 (*p* < .001) for internalizing and 0.51 (*p* < .001) for externalizing problems.

The results from the regression models are shown in Table 2 (for internalizing problems) and Table 3 (for externalizing problems). The initial Model 1 showed a statistically significant association between ACE score and internalizing problems reported by adolescents (β = 0.100, 95% CI [0.060, 0.140]) and mothers (β = 0.168, 95% CI [0.130, 0.207]; Table 2), and externalizing problems reported by adolescents (β = 0.117, 95% CI [0.078, 0.157]; Table 3) and mothers (β = 0.175, 95% CI [0.135, 0.214]). Adjusting for health behaviors, and demographic and birth characteristics (Model 2) resulted in an attenuated but statistically significant association between the ACE score and adolescent adjustment (Tables 2 and 3). Lastly, in Model 3 adjusted for all covariates, including temperament, ACE score was still associated with adolescent (β = 0.063, 95% CI [0.019, 0.107]) and mother-reported (β = 0.120, 95% CI [0.078, 0.163]) internalizing problems (Table 2) and adolescent (β = 0.088, 95% CI [0.045, 0.131]) and mother-reported (β = 0.114, 95% CI [0.072, 0.157]) externalizing problems (Table 3).

**Table 2:** The associations between the ACE score and internalizing problems

Model 1	Adolescent R^2^ = 0.010 (*p* = .013)		Mother/caregiver R^2^ = 0.028 (*p* < .001)	
	β	95% CI	β	95% CI
ACE score	**0.100**	**0.060; 0.139**	**0.168**	**0.131; 0.206**

Model 2	Adolescent R^2^ = 0.023 (*p* < .001)		Mother/caregiver R^2^ = 0.062 (*p* < .001)	
	β	95% CI	β	95% CI
ACE score	**0.066**	**0.022; 0.110**	**0.120**	**0.077; 0.164**
Child sex^male^	-0.034	-0.072; 0.005	0.028	-0.010; 0.065
Low birth weight (< 2,500 g)	0.030	-0.008; 0.068	0.012	-0.027; 0.050
Maternal age	0.006	-0.041; 0.052	**0.070**	**0.024; 0.116**
Parity ^nulliparous^	0.017	-0.032; 0.065	**0.115**	**0.066; 0.164**
Maternal education	-0.030	-0.079; 0.019	0.025	-0.022; 0.071
Maternal prenatal depressive symptoms	**0.048**	**0.003; 0.092**	**0.064**	**0.017; 0.111**
Alcohol use in early to mid-pregnancy	0.016	-0.027; 0.059	**0.056**	**0.015; 0.098**
Smoking in pregnancy ^ex−smokers^	0.008	-0.036; 0.053	-0.056	-0.101; -0.012
Smoking in pregnancy ^smokers^	-0.023	-0.072; 0.026	-0.025	-0.071; 0.022
Financial hardship	**0.055**	**0.014; 0.096**	**0.074**	**0.033; 0.115**
Family structure ^two biological parents^	0.017	-0.028; 0.062	0.007	-0.040; 0.054
ACE score by 3 years	0.037	-0.009; 0.082	**0.065**	**0.018; 0.112**

Model 3	Adolescent R^2^ = 0.036 (*p* < .001)		Mother/caregiver R^2^ = 0.095 (*p* < .001)	
	β	95% CI	β	95% CI
ACE score	**0.063**	**0.019; 0.107**	**0.120**	**0.077; 0.163**
Child sex^male^	-0.035	-0.073; 0.003	0.025	-0.012; 0.062
Low birth weight (< 2,500 g)	0.025	-0.013; 0.064	0.004	-0.034; 0.042
Maternal age	0.002	-0.045; 0.048	**0.063**	**0.018; 0.109**
Parity ^nulliparous^	**0.012**	**0.036; 0.061**	**0.108**	**0.059; 0.156**
Maternal education	-0.039	-0.088; 0.010	0.007	-0.040; 0.054
Maternal prenatal depressive symptoms	0.039	-0.006; 0.084	**0.048**	**0.002; 0.094**
Alcohol use in early to mid-pregnancy	0.013	-0.029; 0.056	**0.053**	**0.012; 0.094**
Smoking in pregnancy ^ex−smokers^	0.005	-0.040; 0.049	**-0.057**	**-0.101; -0.012**
Smoking in pregnancy ^smokers^	-0.020	-0.069; 0.029	-0.019	-0.066; 0.028
Financial hardship	**0.051**	**0.010; 0.092**	**0.069**	**0.029; 0.110**
Family structure ^two biological parents^	0.018	-0.027; 0.063	0.004	-0.042; 0.051
ACE score by 3 years	0.014	-0.033; 0.060	0.024	-0.023; 0.070
Activity	-0.076	-0.129; -0.023	**-0.082**	**-0.133; -0.031**
Emotionality	**0.102**	**0.061; 0.143**	**0.150**	**0.110; 0.189**
Shyness	-0.049	-0.099; 0.002	0.028	-0.020; 0.075

Statistically significant associations are highlighted inbold.

**Table 3:** The associations between the ACE score and externalizing problems

Model 1	Adolescent R^2^ = 0.014 (*p* = .003)		Mother/caregiver R^2^ = 0.031 (*p* < .001)	
	β	95% CI	β	95% CI
ACE score	**0.117**	**0.078; 0.157**	**0.175**	**0.136; 0.214**

Model 2	Adolescent R^2^ = 0.041 (*p* < .001)		Mother/caregiver R^2^ = 0.087 (*p* < .001)	
	β	95% CI	β	95% CI
ACE score	**0.096**	**0.052; 0.140**	**0.127**	**0.083; 0.170**
Child sex^male^	**0.141**	**0.103; 0.179**	**0.203**	**0.166; 0.239**
Low birth weight (< 2,500 g)	0.019	-0.019; 0.057	0.029	-0.010; 0.068
Maternal age	-0.025	-0.072; 0.022	-0.023	-0.068; 0.021
Parity ^nulliparous^	-0.042	-0.090; 0.007	-0.007	-0.053; 0.039
Maternal education	-0.005	-0.053; 0.043	0.029	-0.019; 0.078
Maternal prenatal depressive symptoms	0.011	-0.035; 0.056	0.039	-0.006; 0.084
Alcohol use in early to mid-pregnancy	0.024	-0.019; 0.067	**0.052**	**0.011; 0.093**
Smoking in pregnancy ^ex−smokers^	0.037	-0.005; 0.079	0.010	-0.032; 0.051
Smoking in pregnancy ^smokers^	0.039	-0.007; 0.085	0.023	-0.022; 0.069
Financial hardship	0.027	-0.014; 0.067	0.039	0.000; 0.078
Family structure ^two biological parents^	-0.006	-0.055; 0.042	0.007	-0.037; 0.052
ACE score by 3 years	0.022	-0.027; 0.140	**0.073**	**0.083; 0.170**

Model 3	Adolescent R^2^ = 0.057 (*p* < .001)		Mother/caregiver R^2^ = 0.137 (*p* < .001)	
	β	95% CI	β	95% CI
ACE score	**0.088**	**0.045; 0.132**	**0.114**	**0.072; 0.157**
Child sex^male^	**0.138**	**0.101; 0.176**	**0.197**	**0.161; 0.232**
Low birth weight (< 2,500 g)	0.019	-0.019; 0.057	0.028	-0.011; 0.066
Maternal age	-0.026	-0.072; 0.021	-0.025	-0.068; 0.018
Parity ^nulliparous^	-0.046	-0.094; 0.002	-0.016	-0.061; 0.030
Maternal education	-0.009	-0.056; 0.039	0.021	-0.027; 0.068
Maternal prenatal depressive symptoms	0.003	-0.042; 0.049	0.022	-0.022; 0.066
Alcohol use in early to mid-pregnancy	0.021	-0.021; 0.064	**0.047**	**0.008; 0.087**
Smoking in pregnancy ^ex−smokers^	0.028	-0.014; 0.069	-0.005	-0.045; 0.035
Smoking in pregnancy ^smokers^	0.034	-0.011; 0.080	0.015	-0.028; 0.059
Financial hardship	0.024	-0.017; 0.064	0.034	-0.005; 0.072
Family structure ^two biological parents^	-0.003	-0.051; 0.045	0.012	-0.032; 0.056
ACE score by 3 years	0.030	-0.020; 0.080	**0.085**	**0.038; 0.133**
Activity	0.029	-0.025; 0.082	**0.079**	**0.029; 0.130**
Emotionality	**0.099**	**0.057; 0.141**	**0.192**	**0.153; 0.231**
Shyness	-0.074	-0.123; 0.025	**-0.083**	**-0.130; -0.036**

Statistically significant associations are highlighted inbold

Separate analysis for the ACE types (Table 4) showed statistically significant associations after FDR correction between adolescent adjustment indicators and physical abuse, emotional abuse, parental mental illness, parental offending, parental divorce or separation, and parental conflict and violence. Emotional abuse was significantly associated with internalizing and externalizing problems reported by both adolescents and their mothers while physical abuse was associated with externalizing problems reported by both reporters and internalizing problems reported by mothers (not statistically significant after correction for adolescent report).

**Table 4:** The associations between the ACE types and adolescent adjustment

	Internalizing problems						Externalizing problems					
	Adolescent			Mother/caregiver			Adolescent			Mother/caregiver		
	β	95% CI	R^2^	β	95% CI	R^2^	β	95% CI	R^2^	β	95% CI	R^2^
Sexual abuse	-0.011	-0.061; 0.039	3.3	0.002	-0.043; 0.048	8.4	0.004	-0.039; 0.047	5.1	0.009	-0.036; 0.053	12.7
Physical abuse	**0.047**	**0.003; 0.090**	3.5	**0.093**	**0.052; 0.134**	9.1	**0.069**	**0.028; 0.109**	5.5	**0.089**	**0.049; 0.130**	13.4
Emotional abuse	**0.070**	**0.027; 0.113**	3.7	**0.097**	**0.055; 0.139**	9.2	**0.063**	**0.021; 0.105**	5.5	**0.059**	**0.017; 0.101**	12.9
Parental mental illness	0.033	-0.009; 0.076	3.4	**0.604**	**0.309; 0.899**	9.0	0.029	-0.012; 0.071	5.2	**0.066**	**0.026; 0.105**	13.0
Parental offending	-0.001	-0.044; 0.043	3.3	**0.055**	**0.014; 0.096**	8.6	0.001	-0.042; 0.043	5.1	0.040	-0.003; 0.082	12.8
Parental divorce or separation	0.030	-0.011; 0.071	3.4	0.024	-0.016; 0.065	8.4	**0.061**	**0.020; 0.102**	5.5	**0.046**	**0.006; 0.087**	12.8
Parental conflict and violence	0.035	-0.006; 0.076	3.4	**0.082**	**0.042; 0.122**	9.0	**0.052**	**0.011; 0.093**	5.3	**0.078**	**0.038; 0.118**	13.2
Parental substance use	0.000	-0.041; 0.042	3.3	-0.015	-0.055; 0.025	8.4	0.008	-0.032; 0.047	5.1	0.018	-0.021; 0.057	12.7

Each ACE type was analyzed separately for each outcome, adjusting for the full set of covariates. All R^2^ are statistically significant at *p* < .001. All R^2^ are in %. Statistically significant associations are highlighted in bold.

### Follow-up analyses

The results of moderation analyses indicated that only the main effects of emotionality and ACE were statistically significant. For mother-reported internalizing problems, both ACE score (β = 0.120, 95% CI [0.077, 0.163]) and emotionality (β = 0.150, 95% CI [0.110, 0.189]) were significant predictors, while their interaction was not (β = 0.007, 95% CI [-0.034, 0.048]). Similarly, for adolescent-reported internalizing problems, significant associations were found for ACE score (β = 0.063, 95% CI [0.019, 0.107]) and emotionality (β = 0.102, 95% CI [0.061, 0.143]), but not for their interaction (β = -0.013, 95% CI [-0.054, 0.028]).

In the case of mother-reported externalizing problems, both ACE (β = 0.114, 95% CI [0.071, 0.157]) and emotionality (β = 0.192, 95% CI [0.153, 0.231]) were again significant, but not their interaction (β = 0.063, 95% CI [-0.032, 0.046]). Lastly, for adolescent-reported externalizing problems, ACE (β = 0.089, 95% CI [0.045, 0.132]) and emotionality (β = 0.099, 95% CI [0.057, 0.141]) were both significant, while the interaction was not (β = -0.013, 95% CI [-0.054, 0.028]).

The results of mediation analyses showed significant direct effects of both emotionality and ACE on all outcomes. However, ACE scores did not significantly mediate the relationship between emotionality and: (a) mother-reported internalizing problems (β = 0.004, *p* = .134), (b) mother-reported externalizing problems (β = 0.003, *p* = .137), (c) adolescent-reported internalizing problems (β = 0.002, *p* = .177), or (d) adolescent-reported externalizing problems (β = 0.063, *p* = .150).

## Discussion

The aim of the current study was to test the associations between ACE and adolescent adjustment. Key contribution of our study is confirming previous findings with a more robust prospective design that includes multiple informants and controls for most known confounders, thereby strengthening causal inference and addressing earlier methodological limitations. Additionally, this study is, to the best of our knowledge, the first one that brings evidence from a population-based longitudinal birth cohort based in Central Europe. The results showed that ACE were prevalent in the studied sample and were prospectively associated with indicators of adjustment in young adolescents above and beyond demographics, birth characteristics, and early childhood temperament.

In the ELSPAC-CZ sample, 69.3% of the children were exposed to at least one ACE between ages 3 and 11; 15.9% of the sample was exposed to four or more ACE. The most common ACE was parental mental illness (39.7%), followed by parental conflict (39.6%). The least common ACE types were parental offending (11.6%) and sexual abuse (5.0%). The prevalence of ACE in ELSPAC-CZ cohort was comparable to a study by Veleminsky and colleagues [32], who collected retrospective data on ACE from 1,681 Czech college students (62.2% reported at least 1 ACE, while 9.9% reported ≥ 4 ACE) and to the prevalence reported in other prospective birth cohorts. For example, 58.8% of children in ALSPAC cohort were exposed to one or more ACE by age 8 [39], 66.9% were exposed to ≥ 1 ACE by age 3 in Millennium Cohort Study (MCS; 16), and 67.2% by age 18 in E-Risk study [20].

Upon further investigation, we found that the high prevalence of parental mental illness was driven by maternal depressive symptoms (score >13 on EPDS), reported once during the target period. Similarly, a high prevalence of maternal mental illness was observed also in ALSPAC cohort with identical operationalization of parental mental illness – the prevalence of maternal mental health problems was 32.3% by age 8 [39] and 52.1% by age 19 [46], suggesting that this finding reflects a general trend rather than an idiosyncrasy of the ELSPAC-CZ sample. Similarly, the high prevalence of parental conflict and violence (37.6%) was driven up by an affirmative answer to the item “Your partner was emotionally cruel to you.” It is possible that the way the item was translated and understood by the participants was related to the reasons this item was so commonly endorsed.

The current study confirmed the established link between ACE exposure and mental health outcomes in a prospective birth cohort study based in Central Europe – ACE were associated with both adolescent- and mother-reported internalizing and externalizing problems. Associations were stronger for mother-reported outcomes, which may partly reflect same-reporter bias. Consistent with prior research, adolescents reported higher levels of both internalizing and externalizing problems compared to mothers, with moderate agreement between reporters [47, 48]. These differences underline the value of including multiple informants for both ACE exposure and mental health outcomes, as each contributes unique and complementary information.

The results support the dose-response relationship of ACE to psychopathology, confirming findings from previous studies. For example, Bevilacqua and colleagues [16] found a positive association between ACE score and internalizing and externalizing problems measured by the SDQ in adolescents from the MCS cohort. Similarly, Rebicova and colleagues [33] found in a cross-sectional sample from the Health Behaviour in School-aged Children (HBSC) study in Slovakia that retrospectively collected ACE were associated with increased internalizing and externalizing problems assessed by the SDQ. Additionally, the positive association between ACE exposure and risk for depression in adolescents was consistently found across three prospective cohorts from the UK [49]. The internalizing and externalizing problems assessed at age 11 may represent an early stage in the developmental cascade of difficulties triggered by cumulative ACE exposure in early childhood. Even small increases in problem behaviors at this age can have downstream consequences, including heightened risk of persistent mental health difficulties, academic underachievement, and social problems in later adolescence [50, 51].

Although the association between ACE and adjustment remained statistically significant in fully adjusted model, the effect of ACE was attenuated. Early childhood emotionality, i.e., a tendency to strongly react even to low-intensity stimuli with negative emotions [52], was the most salient covariate. The results showed that this temperamental dimension was associated with increased adolescent and mother-reported internalizing and externalizing problems. Thus, the findings suggest that the tendency to react with anxiety, depression, or aggression in early adolescence is already present in early childhood, prior to ACE victimization. These findings are consistent with a genetically informed analysis by Baldwin and colleagues [53], who concluded that the increased risk of mental health problems in children exposed to ACE was at least partially due to a pre-existing genetic risk. At the same time, and consistently with previous research [22, 53], the results of the current study showed an additional effect of ACE on the outcomes, independent of pre-existing temperamental vulnerability. Further investigation of the associations between ACE, early childhood emotionality, and adjustment revealed that ACE and emotionality were directly and independently associated with each of the outcomes. There was no evidence of a statistically significant interaction between ACE and emotionality, nor of mediation effects whereby ACE would explain the link between early emotionality and later adjustment. The complex associations between temperament and ACE warrant further investigation in future research.

The effect of ACE on adjustment was driven particularly by exposure to emotional and physical abuse. There is substantial support in the literature for the harmful effects of harsh parenting, characterized by a physical and verbal aggression towards the child [54, 55]. Harsh parenting was found to predict adolescent internalizing problems via its negative impact on self-worth, self-esteem, and through dysfunctional cognitive patterns [56, 57]. The mechanism linking emotional and physical abuse to externalizing problems was proposed to be mainly by normalization of aggressive behaviors [58], and emotional dysregulation that may impact both dimensions of adolescent adjustment [59].

Similarly, and consistent with previous studies [60, 61], parental divorce and separation were associated with externalizing problems similarly as parental conflict and violence that may precede the divorce. The link between parental separation and child maladjustment was reported to be mediated by the negative impact of divorce on the family socioeconomic status and by the increased parental stress [61, 62]. Interestingly, we found an effect of parental separation and conflict on externalizing problems only.

Lastly, we found that parental mental illness was associated with internalizing and externalizing problems but only when they were reported by mothers, not adolescents themselves. Maternal reports of child psychopathology are commonly inconsistent with adolescent reports and maternal mental health was found to be an important source of potential bias [63]. It is possible that the association between parental mental health and child adjustment partially reflected the tendency of mothers reporting worse mental health to perceive greater adjustment difficulties in their children that were not reported by the children themselves.

Our findings underscore the importance of developing strategies both to prevent ACE and to mitigate their long-term impact on health. In line with the Czech Republic’s National Strategy for the Prevention of Child Abuse and Neglect, 2008–2018 [64], relevant recommendations include the implementation of parenting support and education programs, home visiting services, provision of economic and social support for families, and school-based initiatives focused on teaching children about safe relationships. Legal measures to eliminate corporal punishment are also essential components of a comprehensive prevention approach.

### Strengths and limitations

The main strength of the current study is the use of a large longitudinal birth cohort, which allowed for the investigation of the prospective associations between ACE and adolescent adjustment. Detailed data on ACE were collected retrospectively, and the indicators of adolescent adjustment were reported by two reporters to minimize the same-reporter bias. Lastly, we adjusted the analysis for a wide number of early life confounders that occurred before ACE victimization, including early childhood temperament.

Despite its strengths, the present study has several limitations that need to be considered in the interpretation of the results. First, we used a relatively narrow definition of ACE; therefore, it is possible that some traumatic events (e.g., the death of a parent, bullying, etc.) remained undetected by the current ACE measure. Similarly, we cannot rule out additional confounding by unmeasured constructs, for example genetic risk for psychiatric disorders or neurodevelopmental conditions of the children (ADHD, autism spectrum, or learning disorders). Second, although the prevalence estimates align with other studies investigating ACE in the Czech Republic [32], the potential for reporting bias cannot be fully ruled out. This concern is especially relevant for instances of sexual abuse, which were reported solely by mothers. It is possible that some mothers were unaware their child had been sexually abused, leading to underreporting.

Lastly, there was considerable attrition of the ELSPAC-CZ sample over time. Approximately 50% of the participants remained in the study until age 11 as reflected in the difference between the initial and the analytical sample. Sample attrition disproportionately affected less educated parents and families with preexisting risk exposures (such as smoking in pregnancy), which may limit generalizability of the results to this group. Additionally, we cannot rule out that participants with greater exposure to ACE or greater mental health problems were more likely to drop out of the study; therefore, there may be reduced variability in both the predictor and the outcome.

## Conclusions and future directions

This study provides novel evidence from a Central European population-based birth cohort, demonstrating a robust, prospective association between ACE and adolescent adjustment. ACE exposure was prevalent in the ELSPAC-CZ sample and was significantly linked to increased internalizing and externalizing problems, even after adjusting for early childhood temperament and sociodemographic factors. While early emotionality emerged as a key covariate, ACE—particularly emotional and physical abuse—showed an independent association with later adolescent adjustment. Future research should further explore genetic and environmental contributions to the ACE-mental health link with an emphasis on mechanisms mediating the ACE exposure and the outcomes and identifying factors that increase vulnerability either to ACE exposure or adverse health effects of the exposure. The existing research on ACE would also benefit from additional evidence from non-Western samples, particularly large, longitudinal, and representative ones.

## Data Availability

The data that support the findings of this study are available from RECETOX, Faculty of Science, Masaryk University but restrictions apply to the availability of these data, which were used under license for the current study, and so are not publicly available. Data are however available from the authors upon reasonable request and with permission of RECETOX, Faculty of Science, Masaryk University.

## Abbreviations

ACE: Adverse childhood experiences
ALSPAC: Avon longitudinal study of parents and children
ELSPAC: European longitudinal study of pregnancy and childhood
EPDS: Edinburgh postnatal depression scale
HBSC: Health behaviour in school-aged children study
MCS: Millennium cohort study
SDQ: Strength and difficulties questionnaire
